# Extracellular Vesicles: A Potential Biomarker for Quick Identification of Infectious Osteomyelitis

**DOI:** 10.3389/fcimb.2020.00323

**Published:** 2020-07-21

**Authors:** Songyun Deng, Yutian Wang, Shiluan Liu, Te Chen, Yanjun Hu, Guangyan Zhang, Xianrong Zhang, Bin Yu

**Affiliations:** ^1^Department of Orthopaedics, Nanfang Hospital, Southern Medical University, Guangzhou, China; ^2^Guangdong Provincial Key Laboratory of Bone and Cartilage Regenerative Medicine, Nanfang Hospital, Southern Medical University, Guangzhou, China

**Keywords:** osteomyelitis, microorganism identification, extracellular vesicles, bacterial aggregation, infection

## Abstract

Effective management of infectious osteomyelitis relies on timely microorganism identification and appropriate antibiotic therapy. Extracellular vesicles (EVs) carry protein and genetic information accumulated rapidly in the circulation upon infection. Rat osteomyelitis models infected by *Staphylococcus aureus, Staphylococcus epidermidis, Pseudomonas aeruginosa*, and *Escherichia coli* were established for the present study. Serum EVs were isolated 3 days after infection. The size and number of serum EVs from infected rats were significantly higher than those from controls. In addition, bacterial aggregation assay showed that the *S. aureus* and *E. coli* formed large aggregates in response to the stimulation of serum EVs from *S. aureus*-infected and *E. coli*-infected rats, respectively. Treatment of EVs-*S. epidermidis* led to large aggregates of *S. epidermidis* and *E. coli*, whereas stimulation of EVs-*P. aeruginosa* to large aggregates of *S. aureus* and *P. aeruginosa*. To evaluate the changes in EVs in osteomyelitis patients, 28 patients including 5 *S. aureus* ones and 21 controls were enrolled. Results showed that the size and number of serum EVs from *S. aureus* osteomyelitis patients were higher than those from controls. Further analysis using receiver operating characteristic curves revealed that only the particle size might be a potential diagnostic marker for osteomyelitis. Strikingly, serum EVs from *S. aureus* osteomyelitis patients induced significantly stronger aggregation of *S. aureus* and a cross-reaction with *P. aeruginosa*. Together, these findings indicate that the size and number of serum EVs may help in the diagnosis of potential infection and that EVs-bacteria aggregation assay may be a quick test to identify infectious microorganisms for osteomyelitis patients.

## Introduction

Posttraumatic or postoperative osteomyelitis, a common and serious complication in orthopedic trauma, presents a variety of clinical challenges. Clinical data have demonstrated that *Staphylococcus aureus* is a leading pathogen for osteomyelitis, followed by *Staphylococcus epidermidis, Pseudomonas aeruginosa*, and *Escherichia coli* (Jiang et al., 2015; Ma et al., 2018; Fily et al., 2019). Prompt diagnosis, timely identification of causative pathogenic bacteria, and appropriate antibiotics are critical for an effective treatment of osteomyelitis.

In clinical tests for osteomyelitis diagnosis, conventional laboratory parameters are C-reactive protein (CRP), erythrocyte sedimentation rate (ESR), and white blood cells (WBCs) (Stucken et al., 2013; Lin et al., 2016). However, as these biomarkers are non-specific inflammatory markers, it is difficult to distinguish infectious diseases from non-infectious inflammatory conditions. Image examinations such as magnetic resonance imaging and single-photon emission computed tomography/computed tomography can help distinguish the local bone inflammation from systematic inflammation and can refine the infection region clearly (Arican et al., 2019; Mejzlik et al., 2019), but their high costs and intricate operation process limit their application. Most importantly, all the tests aforementioned cannot determine the specific pathogenic bacteria to help choose sensitive antibiotics. Additionally, bacterial diagnosis is limited by a high false-negative rate of conventional cultures (Jiang et al., 2015). Therefore, it remains a great challenge to identify microorganisms quickly and accurately.

Extracellular vesicles (EVs) are a heterogeneous group of nanoscale membrane vesicles (MVs) released by cells and can be further classified as exosomes (<150 nm in diameter), microvesicles (100–1,000 nm), and apoptotic bodies (larger than 1,000 nm) based on their biogenesis, size, and biophysical properties (Gurunathan et al., 2019). As EVs carrying proteins, lipids, and various nucleic acids can be released in response to various stimuli, their role as a key regulator in physiopathological cellular processes in cancer, inflammation, and infection has been intensively explored (Hessvik and LIorente, 2018; Panfoli et al., 2018). Studies indicate that EVs may serve as a valuable tool in diagnostics, prognostics, and therapeutics (Hessvik and LIorente, 2018; Panfoli et al., 2018).

It is found that the number of polymorphonuclear cells-derived EVs is significantly increased in the serum from sepsis patients, probably associated with antibacterial effect of host immune system (Timár et al., 2013; Herrmann et al., 2015) and that EVs in the serum from *S. aureus*-infected patients induce distinct aggregation with *in vitro* addition of exogenous *S. aureus* (Timár et al., 2013; Herrmann et al., 2015). We thus made a hypothesis that serum EVs may have a potential role in differential diagnosis of osteomyelitis. Here, we established rat models of tibial osteomyelitis, which resulted from infection with *S. aureus, S. epidermidis, P. aeruginosa*, and *E. coli*, respectively. We found that the particle size and number of serum EVs from infected rats were all significantly increased compared to controls. We also found that the serum EVs from rats infected by specific bacteria could induce strong aggregation of the corresponding bacteria. Furthermore, we demonstrated the differential diagnostic role of serum EVs from *S. aureus* osteomyelitis patients.

## Methods and Materials

### Ethics Statement

All protocols were conducted in accordance with guidelines for the care and use of human subjects and approved by the Ethics Committee of Nanfang Hospital, Southern Medical University. Written informed consent was obtained from participants prior to inclusion in the study. All the patients were recruited from the Department of Orthopedics at Nanfang Hospital, Southern Medical University, from June to October, 2019. Animal studies were conducted in accordance with the Institutional Animal Care and Use Committee and approved by the Ethics Committee of Nanfang Hospital.

### Bacterial Strains and Preparation of Bacteria

*Staphylococcus aureus, S. epidermidis, P. aeruginosa*, and *E. coli* isolated from the osteomyelitis patients from the Department of Orthopedics, Nanfang Hospital, were identified using PHOENIX 100 (Becton Dickinson Microbiology System, Frankin Lakes, NJ, USA). To prepare bacteria for osteomyelitis animal models or bacterial aggregation assay, an isolated colony from a fresh tryptic soy agar plate was inoculated in 10 mL fresh tryptic soy broth overnight at 37°C with shaking at 180 revolutions/min (rpm). Bacteria were harvested by centrifugation, washed twice with phosphate-buffered saline (PBS), and resuspended in PBS. The concentration of each strain was adjusted to an optical density (OD) of 0.5 at 600 nm, approximately equal to 10^8^ colony-forming units/mL (CFUs/mL), for infection in rat osteomyelitis models and an OD of 1.5 for testing the aggregation action.

### Rat Models of Osteomyelitis

Pathogen-free male Wistar rats aged 8 to 10 weeks (300–350 g) were randomly divided into five groups: one control group with sham operation and four infected groups with *S. aureus, S. epidermidis, P. aeruginosa*, and *E. coli*, respectively. Rat osteomyelitis models were established for this study as Kalteis et al. (2006) described with modifications. Briefly, after the rats were anesthetized with pentobarbital, the rat hind limb was shaved and swabbed with povidone-iodine solution. Next, a parapatellar incision was made to expose the tibial plateau before the tibial medullary cavity was opened and widened with a sterile 18-gauge hollow needle. One hundred microliters of bacterial suspension containing 10^8^ CFUs/mL *S. aureus, S. epidermidis, P. aeruginosa*, or *E. coli* was then injected into the medullary cavity by another 18-gauge sterile hollow needle, the tip of which (1.5 mm in length) was cut off and inserted into the medullary cavity. In the control group, 100 μL PBS was injected with a 1.5-mm needle tip inserted into the medullary cavity. A sterile bone wax was then used to close the medullary cavity. Blood samples were collected 3 days after infection for EV isolation.

### Isolation of Serum EVs From Rat Osteomyelitis Models

Six to ten milliters blood samples were collected into a vacuum blood tube without anticoagulant before centrifuging at 2,000 g for 10 min to separate the serum. Serum samples were processed by centrifugation at 3,000 g for 30 min and filtration through a 5-μm filter (Millex Filter Unit; Millipore, Billerica, MA, USA) to remove cell debris. Extracellular vesicles were isolated from serum samples as Herrmann et al. (2015) described previously. Briefly, the serum samples were transferred to a 10.2 mL Beckman centrifuge tube, which was then filled with filtered Hanks balanced salt solution (HBSS). Then, EVs were isolated by ultracentrifugation at 100,000 g for 1 h at 4°C (Optima L-100 XP; Beckman Coulter, Indianapolis, IN, USA). The isolated EV pellets were resuspended in HBSS to one-fifth of the original serum volume. Isolated EVs were then aliquoted and stored at −80°C to avoid repeated freeze-thaw cycles.

Extracellular vesicles were lysed to evaluate their protein amount, and protein concentration was detected using bicinchoninic acid (BCA) protein assay. Briefly, after 25 μL of 5 × cell lysis buffer was added to 100 μL EVs, the lysate was centrifuged at 12,000 rpm for 5 min under 4°C. Supernatant was collected to quantify protein according to manufacturer's instructions (cat. 23225; Pierce™ BCA Protein Assay Kit; Thermo Fisher Scientific, Rockford, IL, USA). The protein concentrations were adjusted based on the original volume of serum from which the EVs were derived.

### Transmission Electron Microscopy

The morphology of EVs was identified using transmission electron microscopy. Extracellular vesicles were prefixed with 2% paraformaldehyde solution and incubated on carbon-coated copper grids for 20 min at room temperature. After rinsing with PBS for three times, samples were fixed with 1% glutaraldehyde solution for 5 min at room temperature, followed by rinsing with distilled water for 10 times. Samples were then stained with 4% uranyl acetate for 5 min and imaged using a transmission electron microscope (Tecnai G2 Spirit; FEI, Hillsboro, OR, USA).

### Nanoparticle Tracking Analysis

The size distribution and number of EVs were assessed by nanoparticle tracking analysis (NTA) using a Nanosight NS300 system (Nanosight NS300; Malvern Instruments Ltd., Malvern, Worcestershire, UK), which is equipped with a 638-nm laser light source and sCMOS camera. Extracellular vesicles were diluted by 1/20 in HBSS, administered manually into the sample chamber using a syringe. Each sample was measured by three 10-s videos and recorded at cameral level 11. The data were analyzed using NTA software version 3.0. The particle number was adjusted based on the original volume of the EV-derived serum.

### Isolation of MVs From Supernatant of Bacterial Culture

The isolation process of MVs from bacteria was developed following the protocol previously described (Kim et al., 2012). Briefly, overnight cultures of *S. aureus, S. epidermidis, E. coli*, and *P. aeruginosa* were pooled separately and centrifuged at 4,000 *g* for 30 min at 4°C. Their supernatant was filtered using a 0.22-μm syringe filter (Millipore) and further centrifuged at 130,000 g (Optima L-100 XP; Beckman) at 4°C for 2 h. The pellet was resuspended in sterile HBSS to concentrate by 10 times, and the particle number was measured via a Nanosight NS300 system (Nanosight NS300; Malvern Instruments Ltd, Malvern, Worcestershire, UK).

### Bacteria Aggregation Assay

The concentration of *S. aureus, S. epidermidis, P. aeruginosa*, or *E. coli* was adjusted to an OD of 1.5 at 600 nm. Each strain was then stained using a SYTO 9 green fluorescent nucleic acid dye (Invitrogen, Carlsbad, CA, USA) following the manufacturer's protocol. The number of serum EVs from bacteria strain-infected rat was adjusted to 1 × 10^10^ particles/mL based on the particle number detected by NTA. To detect bacteria aggregation, 50 μL EVs at 1 × 10^10^ particles/mL mixed with SYTO 9-stained bacteria in a 10:1 volume ratio were incubated for 15 to 20 min at 37°C. Ten microliters of the EVs-bacteria mixture was applied to the hemocytometer and allowed to stay for 5 min and imaged by a fluorescence microscope (BX53; OLYMPUS, Tokyo, Japan) to visualize bacteria aggregation. For each EVs-bacteria reaction, three random fields were imaged under 400 × magnification. The diameters of bacteria aggregates were measured by ImageJ (National Institutes of Health, Bethesda, MD, USA). The bacterial aggregates larger than 3 μm in diameter were counted as positive ones, the quantification of which was expressed as the percentage of positive aggregates in total particles in the field. A total of five independent experiments were performed for each bacteria-EVs aggregation reaction.

### Sodium Dodecyl Sulfate-Polyacrylamide Gel Electrophoresis and Coomasie Brilliant Blue (CBB) Staining

To investigate whether bacterial MVs were a component of serum EVs isolated from infected rat, proteins in serum EVs from infected rats and in bacteria MVs were analyzed with sodium dodecyl sulfate-polyacrylamide gel electrophoresis (SDS-PAGE) (10% resolving gel), and the gel was subsequently stained with CBB G-250. Protein components of EVs and MVs were distinguished according to their different patterns of bands; 7 μg/lane protein was loaded and separated with 10% SDS-PAGE. After electrophoresis, the gel was fixed in fixing solution (50% methanol and 10% glacial acetic acid) for 6 h before soaking in staining solution (0.1% Coomassie Brilliant Blue R-250, 50% methanol, and 10% glacial acetic acid) for 20 min with gentle agitation. Finally, excess staining was eluted with destaining solution (40% methanol and 10% glacial acetic acid). The gel was photographed for further analysis.

### Aggregation of Bacteria With EVs From Neutrophils

To evaluate the possible cellular origin of EVs, we prepared neutrophilic granulocytes from whole blood using a Percoll kit (P8370; Solarbio, Beijing, China) following the manufacturer's instructions. Briefly, 2 mL of rat blood was carefully overlaid onto a three-layer Percoll gradient (75, 65, and 55%) and centrifuged at 1,500 g for 30 min at 4°C. The layer of neutrophilic granulocytes was carefully pipetted, and the cells were washed twice with HBSS. Cell concentration was adjusted to 1 × 10^7^ cells/mL. Bacteria aggregation was performed according to the method previously described (Timár et al., 2013). Briefly, 50 μL opsonized bacteria at 1 × 10^8^ CFUs/mL were added into 450 μL cell suspension and cultured at 37°C for 20 min. Then culture supernatant was collected for preparation of EVs. Finally, the prepared EVs were cocultured with corresponding bacteria for aggregation test.

### Analysis of Serum EVs From Osteomyelitis Patients

Twenty-eight osteomyelitis patients and 21 controls were included in this study. The diagnosis of osteomyelitis was based on the following confirmatory criteria previously described (Morgenstern et al., 2018): supportive histopathological tests, fistula with communication to a bone or an implant, pathogens identified by culture from at least two separate sites in deep tissue, or an implant. Patients who had undergone internal fixation of fracture but finally healed were enrolled as controls. Patients and controls with a history or presence of another infectious disease, diabetes, autoimmune disease, and severe systemic disease were excluded.

Five-milliliter blood samples were collected for isolation of EVs in a procedure as aforementioned. The particle size and number of EVs were assessed by NTA as well. To evaluate the bacteria aggregation effect of EVs from *S. aureus* osteomyelitis patients, the concentration of *S. aureus, S. epidermidis, P. aeruginosa*, or *E. coli* was adjusted to an OD of 1.5 at 600 nm before staining with SYTO 9. Next, 50 μL EVs at 1 × 10^10^ particles/mL were cocultured respectively with SYTO 9-stained *S. aureus, P. aeruginosa, E. coli*, and *S. epidermidis* in a 10:1 volume ratio for 15 to 20 min at 37°C. Quantification of bacterial aggregates was performed as described in the section, Bacteria Aggregation Assay.

### Statistics

Quantitative values are presented as the mean ± SE. Multiple comparisons were assessed by one-way analysis of variance with least significant difference tests. Means between controls and osteomyelitis patients were compared by independent Student *t*-test. Paired *t-*test was used to analyze the aggregation effect of EVs secreted by neutrophils on bacteria. Receiver operating characteristic (ROC) curves were calculated to evaluate the diagnostic efficacy of osteomyelitis biomarkers. SPSS 22.0 was used for statistical analyses (SPSS, Inc., Chicago, IL, USA). *P* < 0.05 was considered to be statistically significant.

## Results

### Characterization of Serum EVs From Osteomyelitis Rats

**Figure 1:**
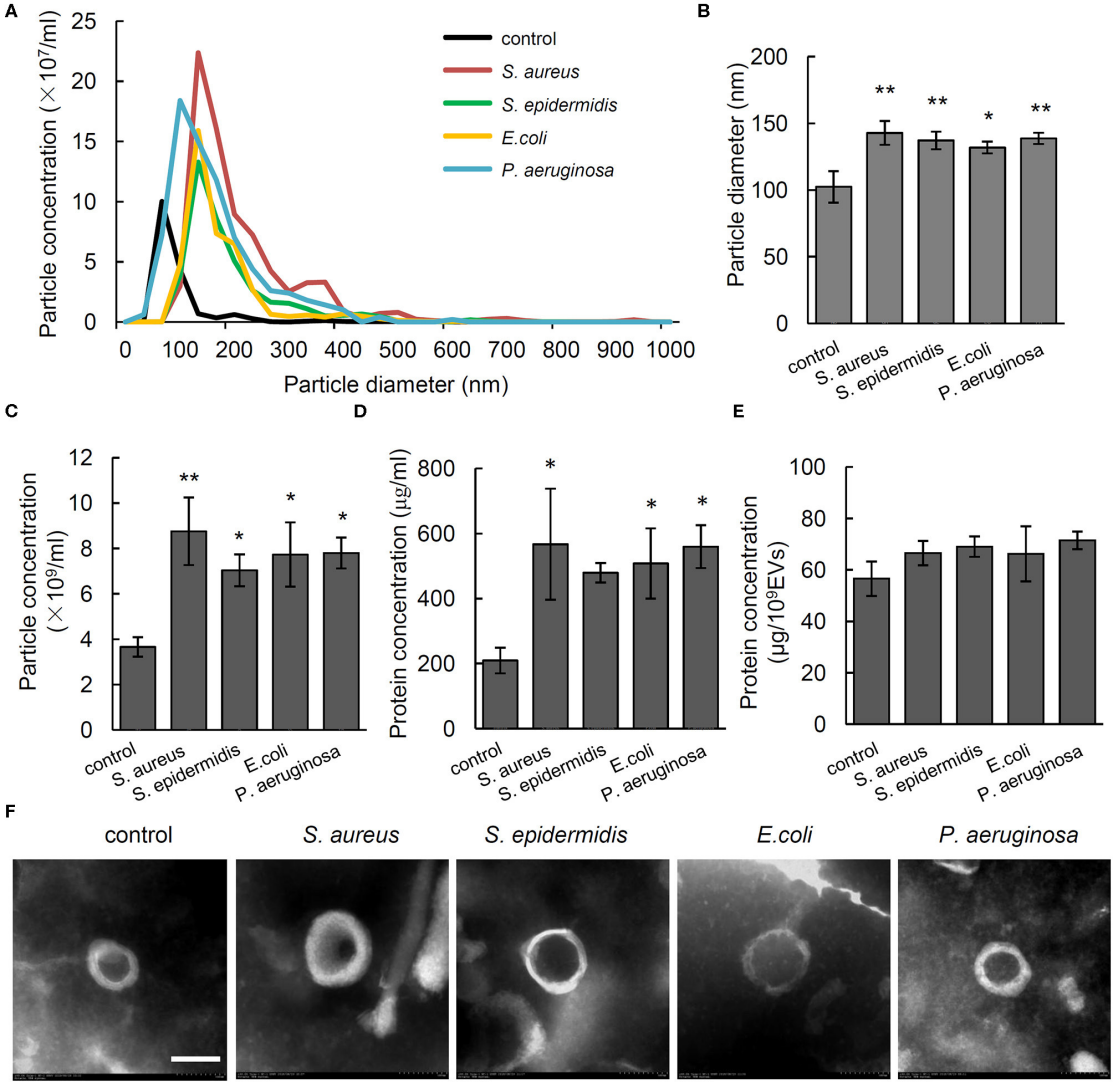
Characterization of serum extracellular vesicles (EVs) from bacteria-infected rats and controls. **(A)** Representative nanoparticle tracking analysis image of serum EVs from *Staphylococcus aureus*-, *Staphylococcus epidermidis*-, *Pseudomonas aeruginosa*-, and *Escherichia coli*-infected rats. The *x* axis is set to scale the size of EVs, and the *y* axis is set to scale the number of EVs. **(B,C)** Quantification of the particle diameter **(B)** and number **(C)** of serum EVs. *n* = 4/group *P < 0.05, **P < 0.01. **(D)** Quantification of protein concentration of EVs in microgram per microliter of serum it derived. *n* = 4/group, *P < 0.05, **P < 0.01. **(E)** Quantification of protein concentration of EVs in microgram per 10^9^ EVs. *n* = 4/group. **(F)** Representative transmission electrical microscopy image of serum EVs from. Scale bar, 100 nm.

To investigate the effect of different bacteria on *in vivo* formation of EVs, the particle size and number of serum EVs from control rats and osteomyelitis rats were evaluated using NTA. We found a significantly increased particle size and number of serum EVs from the rats infected by *S. aureus, S. epidermidis, E. coli*, or *P. aeruginosa* compared with that from control rats (Figures 1A–C). Specifically, the diameter and total number of serum EVs from control rats were, respectively, 102.35 ± 11.84 nm and (3.66 ± 0.43) × 10^9^ particles/mL, but *S. aureus, S. epidermidis, E. coli*, or *P. aeruginosa* infection increased the diameter significantly to 142.78 nm (*P* = 0.002), 137.13 nm (*P* = 0.006), 131.85 nm (*P* = 0.017), and 138.68 nm (*P* = 0.005), respectively, and the number increased significantly to 8.76 × 10^9^ particles/mL (*P* = 0.003), 7.03 × 10^9^ particles/mL (*P* = 0.036), 7.73 × 10^9^ particles/mL (*P* = 0.014), and 7.80 × 10^9^ particles/mL (*P* = 0.013), respectively (Figures 1B,C).

To investigate the association between particle size and number of EVs and amount of protein in EVs, we detected protein concentration in EVs per microliter of serum. Results showed that compared with that in the serum EVs from control rats (209.36 ± 39.36 μg/mL), the protein concentrations were significantly higher in the serum EVs from the rats infected by *S. aureus* (567.22 ± 170.81 μg/mL, *P* = 0.02), *E. coli* (507.56 ± 108.12 μg/mL, *P* = 0.047), and *P. aeruginosa* (559.67 ± 66.04 μg/mL, *P* = 0.022). No such a difference was observed when the value was adjusted to microgram per 1 × 10^9^ EVs (Figures 1D,E). As shown in Figure 1F, electron microscopy of negatively stained EVs showed cup-shaped MVs at 100 to 200 nm in diameter. The above data indicate that the increased number of EVs particles rather than the increased size is associated with up-regulated level of proteins in serum EVs.

### Stimulation of Bacterial Aggregation by Serum EVs From Osteomyelitis Rats

**Figure 2:**
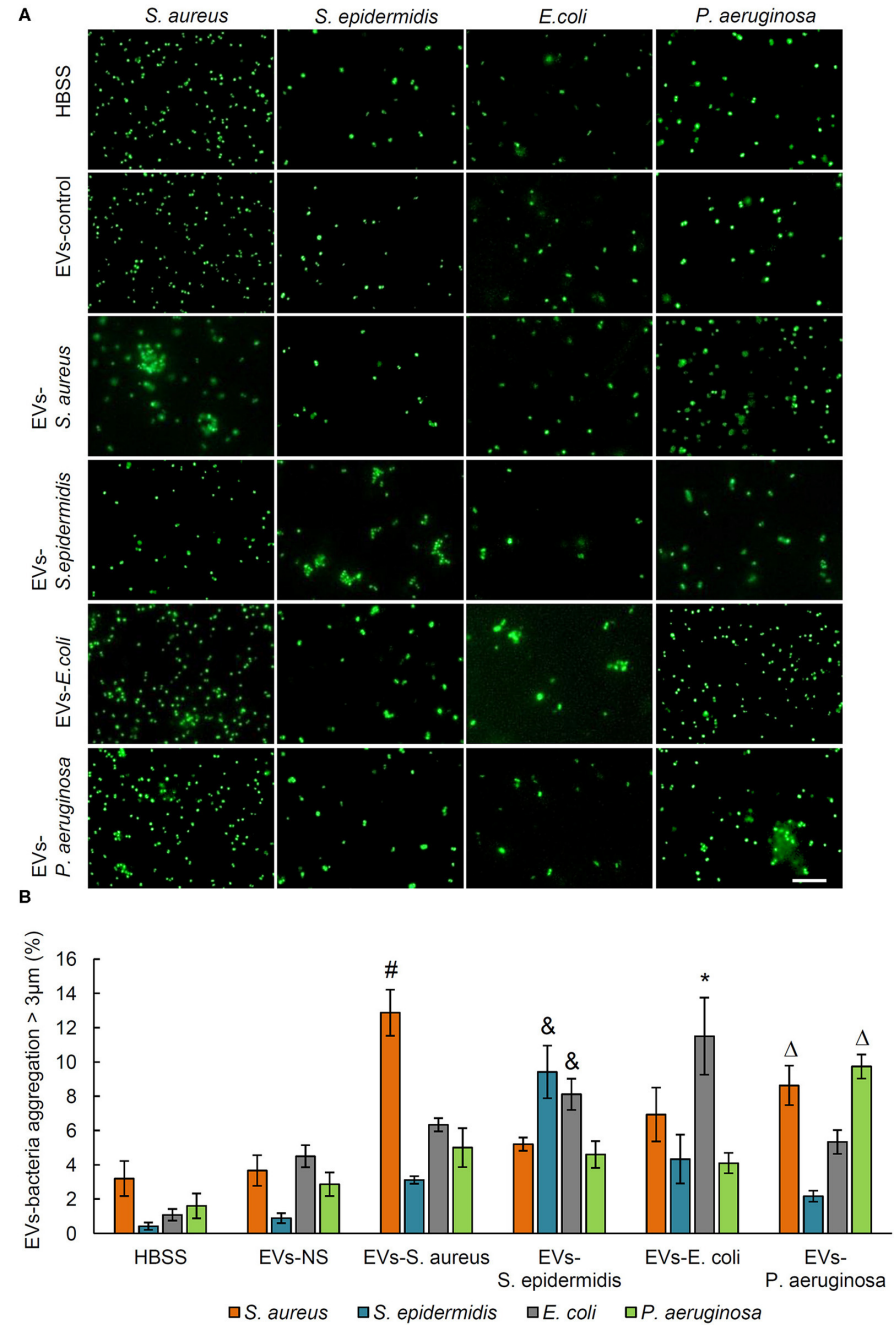
Aggregation activity of *Staphylococcus aureus, Staphylococcus epidermidis, Escherichia coli*, and *Pseudomonas aeruginosa* by treating with serum EVs from bacteria-infected rats and controls. **(A)** Representative fluorescence images of aggregation of *S. aureus, S. epidermidis, E. coli*, and *P. aeruginosa* in response to serum EVs. Scale bar, 10 μm. **(B)** Quantification of EVs-bacteria aggregation with the diameter larger than 3 μm. ^#^*P* < 0.01 vs. aggregation activity of *S. epidermidis, E. coli*, and *P. aeruginosa*. ^&^*P* < 0.01 vs. aggregates of *S. aureus* and *P. aeruginosa*. *P < 0.05 vs. aggregates of *S. aureus, S. epidermidis*, and *P. aeruginosa*. ^Δ^*P* < 0.01 vs. aggregates of *S. epidermidis* and *E. coli*.

The bacteria aggregation activity of EVs from control and bacteria-infected rats was evaluated by incubating EVs with SYTO 9-stained *S. aureus, S. epidermidis, E. coli*, and *P. aeruginosa*. Bacterial aggregation was quantified by counting aggregates larger than 3 μm in diameter using fluorescent microscopy (Figure 2A). As shown by quantitative data in Figure 2B, in the serum EVs from *S. aureus*-infected and *E. coli*-infected rats, massive EVs-bacteria aggregates were observed when EVs were incubated with *S. aureus* and *E. coli*. In response to the treatment of serum EVs from *S. epidermidis*-infected rats, aggregates of *S. epidermidis* and *E. coli* were significantly more than those of *S. aureus* or *P. aeruginosa*. The serum EVs from *P. aeruginosa*-infected rats led to large aggregates of *S. aureus*, as well as those of the *P. aeruginosa*. It is noticeable that the serum EVs from the osteomyelitis rats infected with each of the four strains of bacteria led to large aggregates of a corresponding strain of bacteria with the exception of *E. coli*.

**Figure 3:**
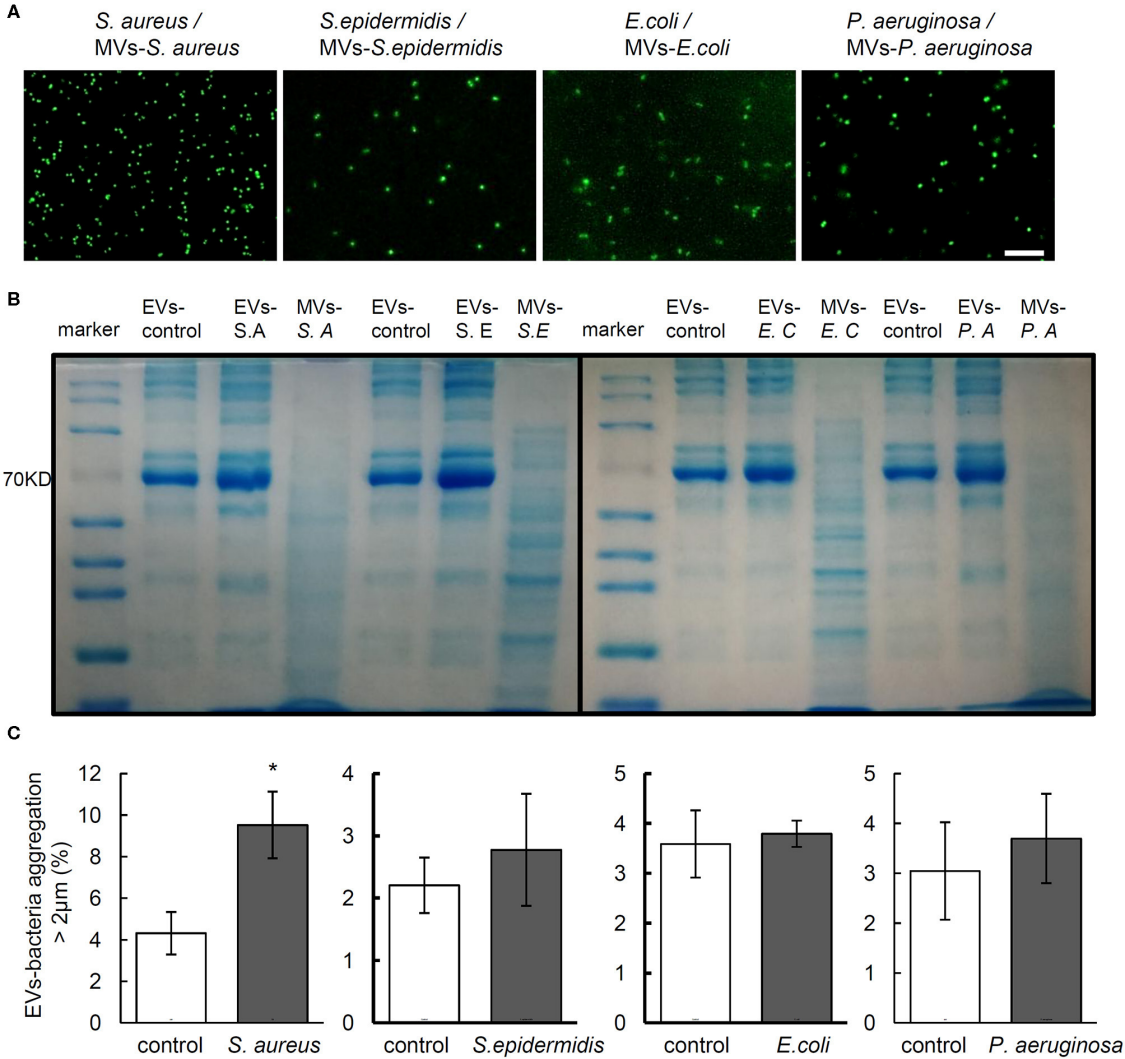
The components EVs but not the MVs mediate bacterial aggregation. **(A)** Aggregation activity of *Staphylococcus aureus, Staphylococcus epidermidis, Escherichia coli*, and *Pseudomonas aeruginosa* with MVs from corresponding bacteria. Scale bar, 10 μm. **(B)** Representative images of Coomasie brilliant blue staining for proteins of serum EVs from control and infected rats and MVs from each bacterium. **(C)** Aggregation activity of *S. aureus, S. epidermidis, E. coli*, and *P. aeruginosa* with EVs from neutrophils infected by corresponding bacteria, respectively. *N* = 3/group, *P < 0.05.

Now that bacterial cells release MVs during host-microbe interactions (Haurat et al., 2015), MVs-bacteria aggregation assay was performed with MVs harvested from the supernatant of bacterial culture to evaluate whether MVs from bacteria may stimulate aggregation of the same bacteria. Results showed that the MVs from the supernatant of bacterial culture failed to induce aggregation of the corresponding bacteria (Figure 3A). To further investigate whether MVs might have been mixed in the serum EVs isolated from the rats infected by different bacteria strain, we separated EV protein from the serum of non-infected and bacteria-infected rats as well, and MVs from the supernatant of *in vitro* bacteria culture using SDS-PAGE, followed by CBB staining to show patterns of protein bands. As shown in Figure 3B, the patterns of serum EV protein from the rats infected by four strains of bacteria were similar to those from the control rats, but the amount of protein close to 70 KD in the EVs from rats infected by each strain of bacteria was much higher than that from control rats. Interestingly, we found that all the band patterns of EVs-*S. aureus*, EVs-*S. epidermidis*, EVs-*E. coli*, and EVs-*P. aeruginosa* were different from those of MVs-*S. aureus*, MVs-*S. epidermidis*, MVs-*E. coli*, and MVs-*P. aeruginosa*, respectively. The above data indicate that aggregation reaction of bacteria-EVs is mainly activated by cellular components from rats infected by each strain of bacteria rather than from bacterial MVs.

### *Staphylococcus aureus* Aggregation Induced by EVs From Neutrophils

It is reported that EVs from neutrophils exposed to *S. aureus* may stimulate EVs-bacteria aggregation (Herrmann et al., 2015). To investigate the possible effect of EVs from neutrophils on bacterial aggregation, EVs were harvested from the supernatant of neutrophils infected by *S. aureus, S. epidermidis, E. coli*, and *P. aeruginosa* and cocultured with each of the above bacteria, respectively, whereas EVs from PBS-treated neutrophils harvested as controls. As shown in Figure 3C, only the EVs of neutrophils infected by *S. aureus* induced significant aggregation of *S. aureus*, whereas no significant bacterial aggregation was observed in cases of the other 3 bacteria.

### Characterization of Serum EVs From Osteomyelitis Patients

**Figure 4:**
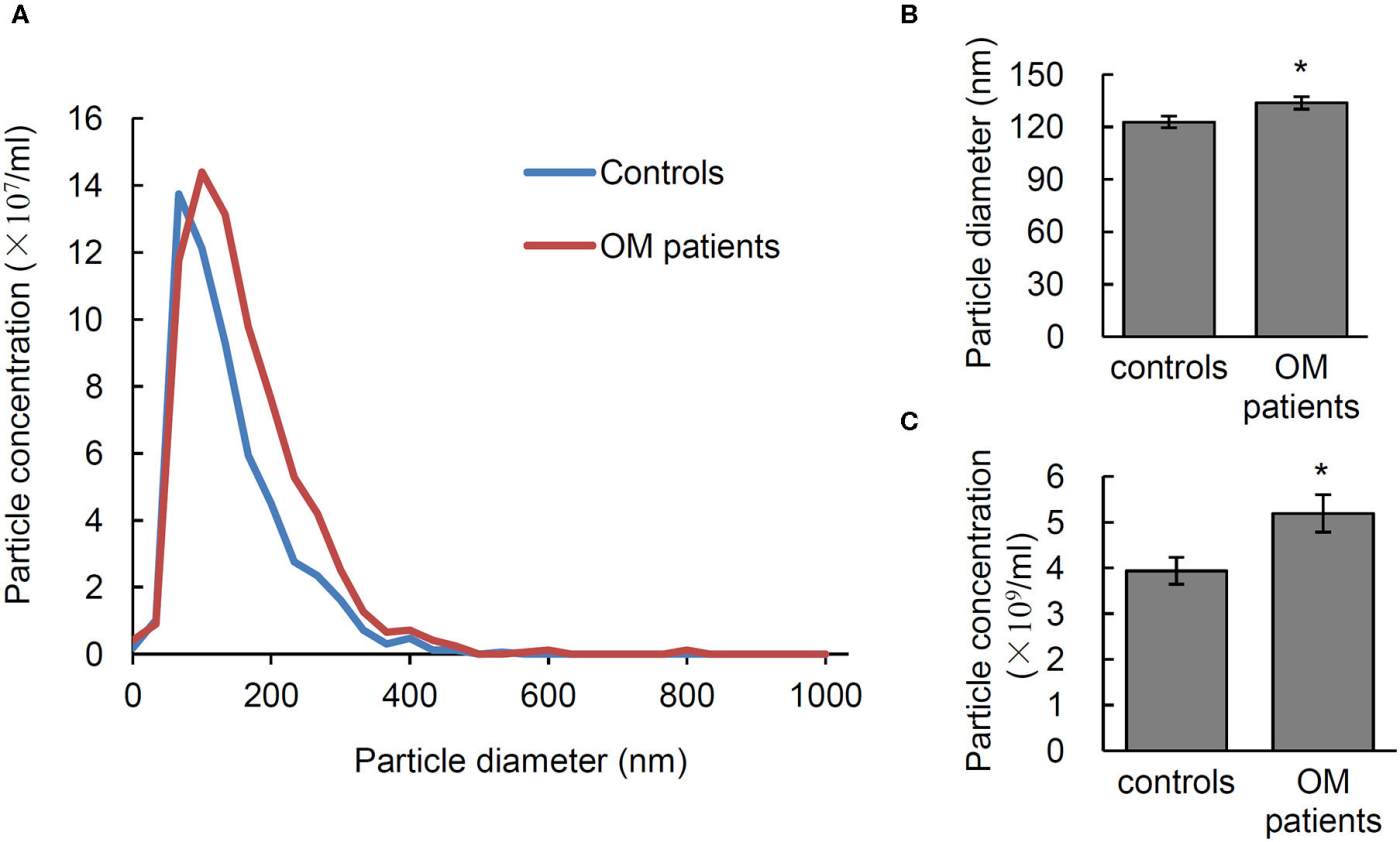
Characterization of serum EVs from osteomyelitis patients. **(A)** Representative nanoparticle tracking analysis (NTA) image of serum EVs. Quantification of the particle diameter **(B)** and number **(C)** of serum EVs from osteomyelitis patients and controls. *P < 0.05.

In order to test the diagnostic potential of EVs in infectious osteomyelitis, the particle size, and number of EVs from 28 osteomyelitis patients and 21 controls were analyzed using NTA. Significant differences were shown in size and number of EVs between osteomyelitis and control patients (Figure 4A). Quantitative data showed that the average diameter of EVs from osteomyelitis patients (133.61 ± 3.55 nm) was significantly larger than that from controls (122.82 ± 3.33 nm, *P* = 0.037) (Figure 4B). The number of EVs from osteomyelitis patients [(5.19 ± 0.41) × 10^9^ particles/mL] was also significantly higher than that from controls [(3.94 ± 0.29) × 10^9^ particles/mL, *P* = 0.024] (Figure 4C). It took less than 4 h to have the particle size and number of EVs determined since collection of blood samples.

### Bacterial Aggregation Activity of EVs From Osteomyelitis Patients

For the 28 osteomyelitis patients, pathogenic microorganisms were identified by positive bacterial culture and PHOENIX 100 (Becton Dickinson Microbiology System). The positive rate of bacterial culture was 13/28. Among those 13 patients with bacterial positive culture, infection by single pathogenic bacteria was observed in eight patients, five of whom were infected by *S. aureus*. Another five patients were infected by more than two strains of bacteria, whereas the other 15 patients had a negative culture.

**Figure 5:**
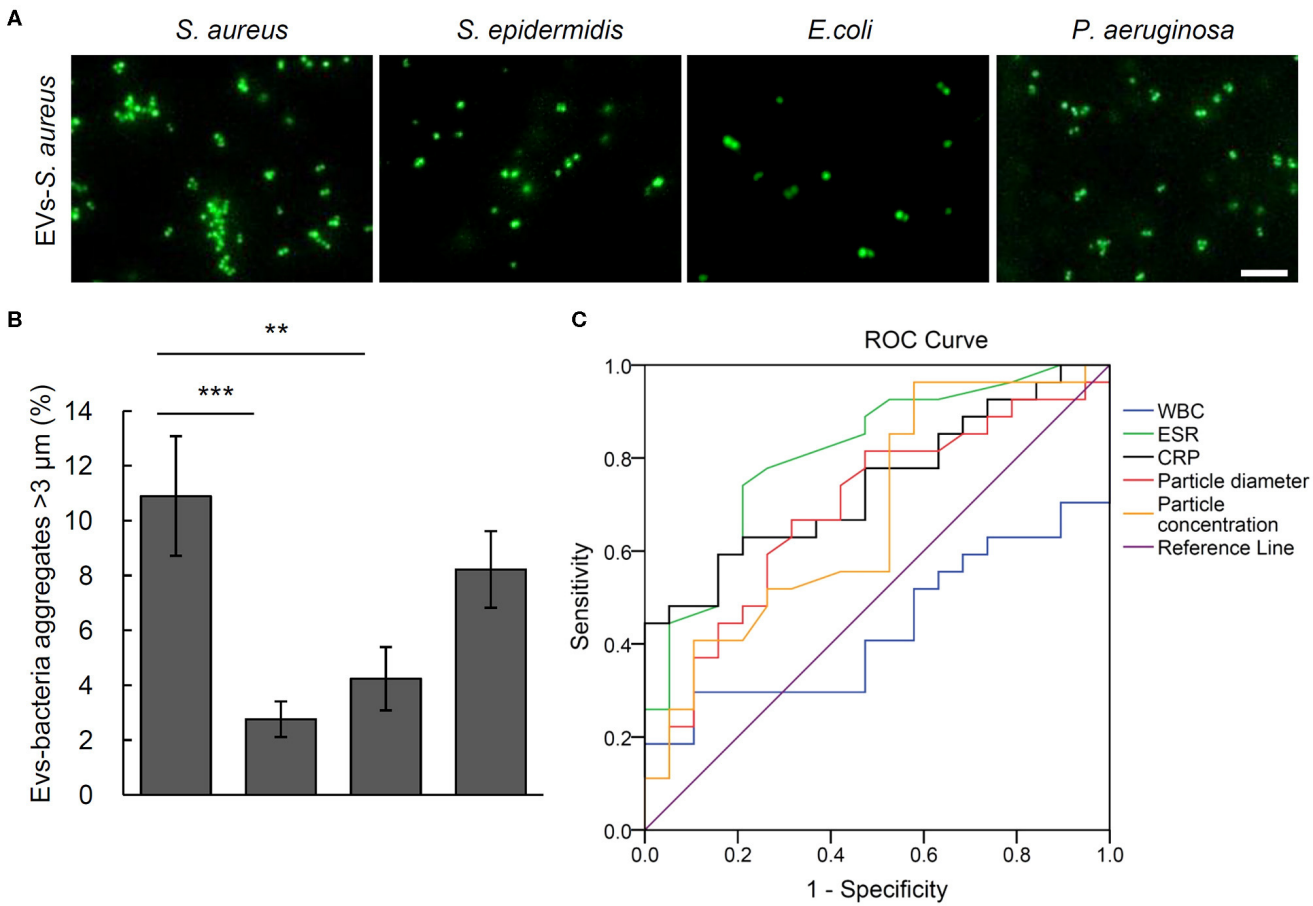
Aggregation activity *Staphylococcus aureus, Staphylococcus epidermidis, Escherichia coli*, and *Pseudomonas aeruginosa* with EVs from *S. aureus* osteomyelitis patients. **(A)** Representative fluorescent images of bacteria aggregation. Scale bar, 10 μm. **(B)** Quantitative analysis of EVs-bacteria aggregates with diameter larger than 3 μm. **P < 0.01, ***P < 0.001. **(C)** Analysis of receiver operating characteristic curves for the diagnostic efficacy of particle diameter of EVs, particle concentration of EVs, and commonly used biomarkers including C-reactive protein (CRP), erythrocyte sedimentation rate (ESR), and white blood cell count (WBC).

To testify the potential of aggregation activity to identify the pathogenic bacteria for osteomyelitis patients, the bacterial aggregation was evaluated of the EVs from the five *S. aureus* osteomyelitis patients. Results showed that serum EVs from *S. aureus* osteomyelitis patients had an aggregation rate of (10.90% ± 2.18%) for *S. aureus* (2.76% ± 0.65%), for *S. epidermidis* (*P* = 0.001 vs. *S. aureus*) and (4.24 %± 1.15%) for *E. coli* (*P* = 0.005 vs. *S. aureus*); however, the aggregation rate for *P. aeruginosa* was (8.22% ± 1.40%) (*P* = 0.211 vs. *S. aureus*), indicating a weak cross-reaction between serum EVs from *S. aureus* osteomyelitis patients and *P. aeruginosa* (Figures 5A,B). It took less than 18 h (including 16 h for bacterial recovery) to finish the bacterial aggregation assay since collection of blood samples.

### Extracellular Vesicles as a Potential Biomarker in Quick Diagnosis of Osteomyelitis

Receiver operating characteristic curves and the corresponding area under the curve (AUC) values were calculated to evaluate the diagnostic efficacy of such biomarkers commonly used for osteomyelitis as WBCs, ESR, and CRP, as well as the size and number of EVs. The closer AUC is to 1, the better the diagnostic efficacy. Erythrocyte sedimentation rate and CRP showed significantly diagnostic AUC values at 0.829 ± 0.066 (*P* = 0.001) and 0.767 ± 0.073 (*P* = 0.005), respectively, but WBCs did not at 0.438 ± 0.089 (*P* = 0.516). As the AUC value of EVs number was 0.662 ± 0.089 (*P* = 0.088), it showed little value in distinguishing osteomyelitis patients from controls, but ROC analysis of the particle size of EVs showed an AUC value of 0.722 ± 0.079 (*P* = 0.019). Further analysis indicated that the best diagnostic threshold value should be 136.95 nm, with a sensitivity of 46.2% and specificity of 93.3% (Figure 5C).

## Discussion

The present study found a definite association between osteomyelitis and the size and number of serum EVs. In addition, serum EVs from rats infected by *S. aureus, S. epidermidis, P. aeruginosa*, or *E. coli* trigger intense aggregation of a corresponding strain of bacteria. Because ~1 week is required for the growth and identification of microorganisms using conventional bacterial culture (Lesens et al., 2011), the significant clinical value of our work is that serum EVs might be a potential biomarker for a quick diagnostic test for osteomyelitis patients to identify the disease and possible infectious microorganisms as well, despite possible cross-reaction induced by other bacteria.

Consistent with a finding that reported EVs accumulate rapidly in the circulation upon infection (Singh et al., 2012; Schorey et al., 2015), we found that the size and number of serum EVs from *S. aureus*–, *S. epidermidis*–, *P. aeruginosa*–, and *E. coli*–infected rats and from osteomyelitis patients increased significantly. However, ROC analysis showed only the particle size was a potential diagnostic marker for osteomyelitis patients. The discrepancy in the effect of EVs between animal models and human patients may be attributed to antibiotic pretreatment and different stages of osteomyelitis. In the present study, all the patients were in an acute stage of chronic osteomyelitis, whereas the animals were on day 3 after acute osteomyelitis infection. It is likely that the particle size and number may be sensitive and specific markers for diagnosis of acute osteomyelitis rather than for acute stage of chronic osteomyelitis. As early diagnosis of acute osteomyelitis is often challenging but critical for timely treatment to minimize bone destruction, it is particularly significant to use particle size and number as biomarkers in diagnosis of acute osteomyelitis.

It is reported that *S. aureus* infection can induce formation and secretion of EVs from neutrophilic granulocytes, and in turn, these EVs demonstrate a definitive ability to stimulate aggregation of *S. aureus ex vivo* (Timár et al., 2013; Herrmann et al., 2015). Consistently, we also found that EVs from neutrophils induced bacterial aggregation. Further, besides specific bacterial aggregation activity of serum EVs from bacteria-infected rats, we found that the serum EVs from patients with *S. aureus* osteomyelitis also induced aggregation of *S. aureus* and a weak cross-reaction of *P. aeruginosa*. Our findings point to a potential role of EVs-bacteria aggregation assay as a quick test to identify possible pathogens for osteomyelitis.

In addition to host components, pathogen-derived components have also been found on EVs after infection (Schorey et al., 2015). Moreover, secretion of MVs is a conserved process from microorganisms to multicellular organisms. Studies demonstrate that Gram-positive and Gram-negative bacteria can produce a variety of MVs, an important role in eliminating competing organisms, antibiotic resistance, and pathological functions in the whole infection process (Lee et al., 2009; Kulp and Kuehn, 2010). Our *ex vivo* and *in vitro* results demonstrate that MVs produced from bacteria cannot induce aggregation of bacteria, and the protein patterns of EVs from infected rats are different from those of MVs, suggesting that the bacterial aggregation induced by the serum EVs may be produced mainly by the infected host cells.

In conclusion, further clinical studies are needed to confirm our chief finding that serum EVs may be used for diagnosis of acute osteomyelitis and to identify the pathologic microorganisms, which is much more rapid than bacterial cultures. However, our study had several limitations. First, because osteomyelitis patients infected by *S. epidermidis, E. coli*, and *P. aeruginosa* were not available for the present study, bacterial aggregation assays of the EVs from them need to be carried out in the future study. Second, although we found the components of EVs mediating bacterial aggregation were from host cells but not from the bacteria, the composition of EVs was not defined. Third, cross-reaction of EVs-bacteria aggregation is of particular concern for its further application; therefore, determination of the essential component in EVs that mediates bacterial aggregation is necessary to help in the development of sensitive and specific methods to define specific pathogenic microorganisms in osteomyelitis patients. Further studies, both laboratory and clinical, are also warranted to determine and improve the accuracy of EVs-bacteria aggregation in identifying the causative organisms for osteomyelitis.

## Data Availability Statement

All datasets generated for this study are included in the article/supplementary material.

## Ethics Statement

The studies involving human participants were reviewed and approved by Ethics Committee of Nanfang Hospital of Southern Medical University. Written informed consent to participate in this study was provided by the participants' legal guardian/next of kin. The animal study was reviewed and approved by Ethics Committee of Nanfang Hospital of Southern Medical University.

## Author Contributions

SD and XZ designed the experiments and analyzed the data and wrote the manuscript. SD, YW, and SL performed the experiments and analyzed the data. TC, YH, and GZ performed the experiments. XZ and BY supervised the experiments, revised, and approved the manuscript. All authors contributed to the article and approved the submitted version.

## Conflict of Interest

The authors declare that the research was conducted in the absence of any commercial or financial relationships that could be construed as a potential conflict of interest.
